# Oxidative Stress in DNA Repeat Expansion Disorders: A Focus on NRF2 Signaling Involvement

**DOI:** 10.3390/biom10050702

**Published:** 2020-05-01

**Authors:** Piergiorgio La Rosa, Sara Petrillo, Enrico Silvio Bertini, Fiorella Piemonte

**Affiliations:** Unit of Muscular and Neurodegenerative Diseases, Bambino Gesù Children’s Hospital, IRCCS, 00146 Rome, Italy; piergiorgio.larosa@opbg.net (P.L.R.); sara.petrillo@opbg.net (S.P.); enricosilvio.bertini@opbg.net (E.S.B.)

**Keywords:** DNA repeat expansion disorders, NRF2, oxidative stress, FXTAS, fragile X syndrome, Friedreich’s ataxia, myotonic dystrophy, spinocerebellar ataxia, Huntington’s disease, spinal and bulbar muscular atrophy

## Abstract

DNA repeat expansion disorders are a group of neuromuscular and neurodegenerative diseases that arise from the inheritance of long tracts of nucleotide repetitions, located in the regulatory region, introns, or inside the coding sequence of a gene. Although loss of protein expression and/or the gain of function of its transcribed mRNA or translated product represent the major pathogenic effect of these pathologies, mitochondrial dysfunction and imbalance in redox homeostasis are reported as common features in these disorders, deeply affecting their severity and progression. In this review, we examine the role that the redox imbalance plays in the pathological mechanisms of DNA expansion disorders and the recent advances on antioxidant treatments, particularly focusing on the expression and the activity of the transcription factor NRF2, the main cellular regulator of the antioxidant response.

## 1. Introduction

Microsatellites are stretches of DNA abundantly interspersed in the genome of prokaryotes and eukaryotes [1,2], including humans, where they account for the 3% of the genome [3]. Their structure consists of short, tandemly repeated duplications of 1–6 base pairs, spanning between 20–100 bases [4] and primarily consisting of mono- and dinucleotides, although tri-, tetra-, penta-, and hexa-nucleotides microsatellite classes are present [4,5]. Their location is ubiquitous, occurring both in protein-coding as well as in non-coding DNA regions, with preference for the latter [6], and their functions range among several biological regulatory processes [7], including alternative splicing [8], transcription start/end site selection [9,10], nucleosome packaging [11], and methylation [12]. One of the most peculiar characteristics of microsatellites is their tendency to mutate. While 10^−9^ is the rate of mutations occurring in non-repetitive region of the DNA, microsatellite mutation rate ranges between 10^−2^ and 10^−6^ [13,14]. Because of this, microsatellites are highly polymorphic, as the number of the repeats in a given locus is relatively unstable and frequently varies between individuals [15]. Due to this feature, deregulated microsatellite expansions are widely known to be the triggering cause of many neurological and neuromuscular diseases and, to date, more than 30 disorders are known to be caused by aberrant expansion of repetitive DNA sequences [16]. Although trinucleotides repeats, in particular GAA, CGG, CAG, and CTG, are commonly known to be responsible for the pathologic manifestation of nucleotide expansion disorders, also tetra- and penta-nucleotides expansions are disease causing [17], thus these diseases are collectively classified as disorders of DNA unstable repeat expansions [18]. Some peculiarities join this variegated group of pathologies: (i) The disease-causing expansion repeats are more unstable in the affected population, with a higher tendency to expand or to contract with respect to the polymorphic repeats of normal people, even if contraction events are rarer [16]. This also occurs as the repeats reach the threshold limit between a normal condition and the pathological state. Thus, even in unaffected families, de novo mutations can determine clinical manifestations [19]. (ii) The dynamic changes in the length of expansions are so marked that differences among patients, as well as in different tissues of the same affected proband, are common [16]. (iii) The more the expansions are transmitted from generation to generation, the earlier the disease symptoms appear in the newly affected individuals, a phenomenon known as clinical anticipation [20,21,22]. (iv) In most disorders, the length of repeat expansions influences the phenotype severity of affected individuals [23,24,25,26,27,28,29]. Consequently, different defects for a given disease can be expressed among patients, including onset of symptoms and co-morbidities. For example, the incidence of diabetes in Friedreich’s ataxia (FA) ranges between 8–32% of patients [30,31,32], but the risk of developing is directly correlated to the number of GAA repeats in the frataxin (*FXN*) gene [33]. In the same way, in myotonic dystrophy (DM), patients carrying small CTG repeats (i.e., between 50–99) are asymptomatic or develop mild defects, such as cataracts, while a severe phenotype occurs in patients with 100–200 repeats [34].

Beside these common features, a high grade of diversity characterizes DNA expansion disorders, as the DNA expanded tracts can affect genes encoding proteins with different roles. Therefore, three different classes have been distinguished, by assembling disorders on the basis of which defects arise from expansions. The first class groups the diseases that are determined by a protein loss of function and are inherited by autosomal recessive or x-linked manner [19]. Typical examples are Fragile X (FXS) or FA, where CGG or GAA repeats determine the loss of the expression of the fragile X mental retardation protein (FMRP) and FXN, respectively [35,36]. The second group belongs to disorders characterized by an autosomal dominant inheritance and in which a protein gain of function occurs [18]. PolyQ diseases, for instance, are determined by CAG expansions in the coding region of 9 distinct genes that lead to the formation of glutamine residues in the final peptide of their encoded product [37]. PolyQ tracts exert a toxic effect mainly by causing aberrant nuclear and cytoplasmic protein aggregation and trapping transcription factors [38,39], chaperons, and proteins belonging to the ubiquitin–proteasome system (UPS) [40]. The third group of disorders are characterized by gain of function involving the transcribed RNA. DM1 and DM2 are respectively caused by the aberrant insertion of CTG and CCTG expansion repeats in the 3′ untranslated region (UTR) of dystrophin myotonic protein kinase (*DMPK*) [41] and in the first intron of zinc-finger protein 9 (*ZNF9*) [42]. Similarly, the CGG triplet expansion in *FMR1* gene, ranging between 60–200 triplets, causes the fragile X–associated tremor ataxia syndrome (FXTAS) [43]. The pathogenic activity, both in DM and in FXTAS, lies on of the respective expanded mRNA molecules, which are able to sequestrate RNA binding proteins, such as muscleblind-like (MBNL) proteins in DM1, determining splicing alterations and impairments in protein expression [44,45,46,47].

**Figure 1:**
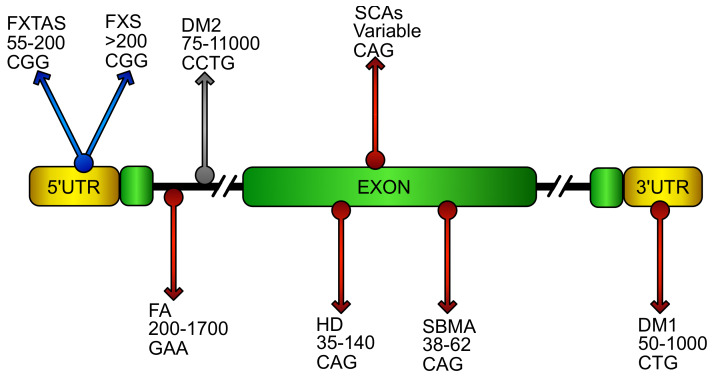
Putative location and sequence of DNA expansions in repeat disorders. Schematic representation of an ideal gene showing DNA repeat expansions that cause diseases. Name of the relative disorder, number of pathogenic repeats, and its sequence are reported in the gene region were the repeats stem in the pathology. The grey arrow represents pathology where oxidative stress has been poorly investigated. Blue arrows characterize diseases with oxidative stress contributions. Red arrows identify pathologies in which NF-E2 p45-related factor 2 (NRF2) involvement has been reported. (FXTAS, fragile X–associated tremor ataxia syndrome; FXS, fragile X syndrome; FA, Friedreich’s ataxia; DM1/DM2 myotonic dystrophy; HD, Huntington’s disease; SCAs, spinocerebellar ataxias; SBMA, spinobulbar muscular atrophy).

Oxidative stress has been widely reported to play a prominent role in neurodegenerative diseases [48,49], including disorders caused by DNA expansion repeats (Figure 1). Here, we report the most recent evidences connecting ROS imbalance and DNA expansion disorders, with particular emphasis to the pathway primarily involved in the regulation of cellular antioxidant response, the NF-E2 p45-related factor 2 (NRF2) signaling pathway.

## 2. Oxidative Stress and Cellular Responses

Oxidative stress occurs when the balance between ROS production and elimination cannot be maintained in the cell, thus determining damage on lipids, proteins, and nucleic acids, ultimately leading to cell death [50,51]. Oxidative stress can be determined by exogenous (i.e., UV light or chemicals) and endogenous factors [52], as mitochondrial metabolism and NADH oxidase activity, the major endogenous ROS sources [53,54]. ROS production and elimination are tightly controlled in the cell, as aerobic organisms use O_2_ molecules in redox reactions needed for energy demands and oxygen byproducts (superoxide anion, O_2_^−^, hydrogen peroxide, H_2_O_2_, hydroxyl radical, HO*) to transduce regulatory signals [55,56,57,58]. Thus, an efficient antioxidant cellular machinery is essential to mitigate adverse effects and to permit a correct signaling cascade modulation. This variety of endogenous defenses consists of antioxidant enzymes, including superoxide dismutases (SODs) [59], catalase [60], glutathione peroxidases (GPXs) [61], and scavenger molecules, such as glutathione (GSH), ubiquinol (CoQ10), uric acid, and others [62].

GSH, in particular, represents the most important endogenous antioxidant for its dual function, as a direct ROS scavenger [63,64] and as cofactor in reactions catalyzed by antioxidant enzymes (e.g., glutathione reductase, GR, GPXs) [65] and in those involved in the elimination of xenobiotics (glutathione-S transferases, GSTs) [66]. The GSH synthesis occurs in 2 steps, the first uses cysteine and glutamate as substrates and is catalyzed by the glutamate cysteine ligase (GCL), while the second, catalyzed by the glutathione synthetase (GSS), binds glycine to the pre-synthesized dipeptide [67,68].

Under physiological conditions, a basal expression of this antioxidant machinery is available in cells. However, to promptly answer to redox imbalance that ranges from mild to high pathologic- induced oxidative stress [69,70], cells evolved the transcription-independent activation of NRF2, able in turn to modulate the expression of several antioxidant enzymes (SODs, catalase, GPXs), to ensure the GSH synthesis (by GCL expression) and to reduce toxic quinones by the action of NAD(P)H: quinone oxidoreductase (NQO1) [70,71,72].

## 3. NRF2 Pathway and Its Regulation

NRF2 is a transcription factor belonging to the cap ‘n’ collar (CNC) basic leucine zipper (bZip) proteins [73,74]. In the nucleus, it dimerizes with the small musculoaponeurotic fibrosarcoma (sMAF) proteins, particularly with F, G, and K isoforms [75,76]. The NRF2-sMAF heterodimer binds to specific 16 base long DNA stretches (the antioxidant responsive elements, ARE), acting as enhancer for gene transcription [77]. NRF2 is able to regulate the expression of at least 250 genes [78] and, besides being the master regulator of cellular antioxidant defense, its activity participates to the modulation of different cellular processes, including metabolism, survival, differentiation, inflammation, mitochondrial biogenesis, and mitophagy [69,78,79,80,81,82,83,84]. For this reason, the NRF2 activity and expression are subjected to a tight and fine-tuned control mechanism, to avoid unwanted gene expression upregulation and, at the same time, determining a fast response in case of need. Upon oxidative stress, the induction of NRF2 occurs by regulating its stability and localization in the cell [72], and modulating the amount of its mRNA transcript [81,85].

Under physiological condition, NRF2 has a short half-life, spanning between 15–40 min, and its cellular localization is restricted to the cytoplasm [86]. Soon after translation, NRF2 interacts with the ubiquitin ligase adaptor KEAP1 (Kelch-like ECH-associated protein 1) that sequesters the transcription factor and mediates its proteasomal degradation [87]. In parallel, free NRF2 can be phosphorylated by the GSK3β kinase, which increases the NRF2 proteasomal-mediated turnover [71,86]. These two mechanisms work in conjunction to regulate NRF2 activity, in response to different cellular cues. Under redox imbalance, the KEAP1–NRF2 interaction is disrupted, as result of ROS-induced conformational changes of KEAP1 [88]. Conversely, the activation of growth factor receptors determines the AKT/PI3K-induced inhibitory phosphorylation of GSK3β, thus allowing NRF2 accumulation [89]. This regulatory system points to the NRF2/ARE axis as one of the most important signaling pathway in cells. Indeed, being ARE sequences implicated in the regulation of more than 1% of human genes [86], impairments of NRF2 signaling network may interfere with multiple cellular processes and determine redox imbalance, a condition commonly encountered in cancers [90,91,92] and in neurological disorders. Re-establishing NRF2 signaling homeostasis can be essential to improve the pathological phenotypes, especially in neurodegenerative diseases [93,94,95].

Recent evidences increasingly highlight a dual role of NRF2 in the diseases’ pathogenesis. In cancer, for instance, the activation of Nrf2 appears correlated with progression and chemo-resistance, and its downregulation has attracted growing attention as alternative cancer therapy [96]. Several studies have clearly demonstrated that the hyper-activation of the NRF2 pathway may create an environment favoring the survival of malignant cells, protecting them against oxidative stress, chemotherapeutic agents, and radiotherapy [97,98]. Indeed, although a transient NRF2 activation in response to stress may be beneficial for health, a persistent induction can confer therapeutic resistance in cancer cells and more aggressive tumorigenicity, leading to poor prognoses in patients. In this light, the inhibition of NRF2 is a promising therapeutic approach in cancer and NRF2 inhibitors are being actively developed [99]. Contrariwise, NRF2 appears inhibited in many neurodegenerative disorders, such as Huntington’s disease, Alzheimer’s disease, amyotrophic lateral sclerosis, multiple sclerosis and FA, where its activation has been proven mitigating pathogenic processes by upregulating antioxidant defenses, decreasing inflammation, and improving mitochondrial function [80,100,101,102,103]. Therefore, a dual face of Nrf2 in cancer and neurodegenerative diseases has to be recognized, making its role in the pathogenesis’ mechanisms even more attractive.

## 4. Oxidative Stress in Loss of Function DNA Expansion Disease

### 4.1. Friedreich’s Ataxia (FA)

(FA) is an autosomal recessive neurodegenerative disease caused by a homozygous GAA trinucleotide repeat expansion in the first intron of the FXN gene, encoding for the mitochondrial FXN protein [36,104]. The GAA repeated expansions cause histones deacetylation and abnormal DNA conformation, leading to decreased mRNA levels and FXN amount [105]. FA is clinically characterized by progressive ataxia, diabetes, cardiomyopathy, skeletal deformations, altered central and peripheral nervous system with lesions in the dorsal root ganglia, dentate nuclei of the cerebellum, corticospinal tracts, and sensory peripheral nerves [106,107,108]. Actually, the FXN function is still unclear, although it is well known to be involved in iron–sulphur cluster biogenesis and in heme biosynthesis. The FXN deficiency increases the mitochondrial iron content, altering activities of iron–sulphur (Fe-S) cluster enzymes in mitochondria and causing oxidative stress in affected tissues [109,110,111]. Oxidative stress is a leading hypothesis in the pathogenesis of FA, since the identification of the gene in 1996 and later supported and confirmed by several studies in human and mouse FA models [112,113,114,115].

**Figure 2:**
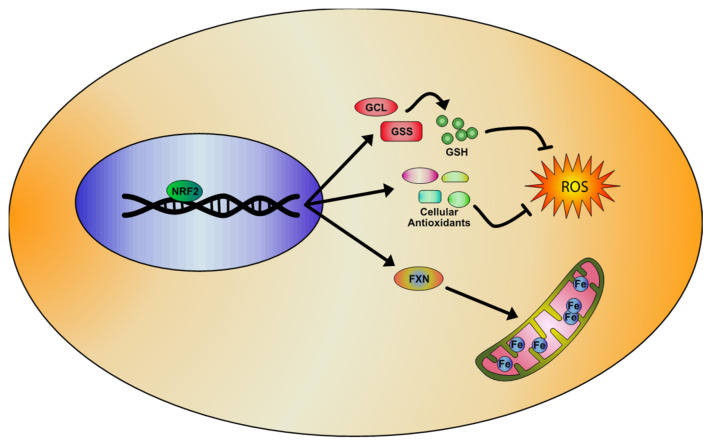
Representative model of the NRF2 signaling pathway activation in Friedreich’s Ataxia (FA), based on literature evidences. NRF2 inducers determine the activation of antioxidant genes transcription and the upregulation of enzymes involved in the regulation of glutathione (GSH) expression, rebalancing the unpaired GSH/GSSG ratio and reducing oxidative stress and lipid peroxidation. Importantly, NRF2 also increases frataxin (FXN) levels, thus partially rescuing the mitochondrial defects observed in FA pathology.

Several studies have demonstrated an impairment of the NRF2 pathway in FA [116,117,118,119] and alterations of systemic redox markers have been evidenced in patients. An increased oxidative damage on nuclear and mitochondrial DNA has been found in peripheral blood cells, together with high levels of plasma malondialdehyde and of urine 8-hydroxy-2-desoxiguanosine [120,121]. A decrease of glutathione levels and of antioxidant enzymes activities (SOD and GST) have been also reported in fibroblasts and in blood of patients [122,123]. Recently, lipid peroxidation and ferroptosis have also been suggested as responsible for the FA pathophysiology [124,125,126]. It is important to note that two ferroptosis-triggering enzymes (GPX4 and cysteine/glutamate transporter system, xC-/xCT) are downstream targets of NRF2 [127]. For all these findings, NRF2 has recently attracted attention for novel therapeutic strategies in FA [72,94,128,129]. Among the main pharmacological NRF2 activators, we can mention the Sulforaphane (SFN), a natural blood–brain-barrier permeable antioxidant, and the dimethyl fumarate (DMF), an ester of fumaric acid recently approved for the treatment of multiple sclerosis [130,131,132,133] and promising for adrenoleukodystrophy [134]. The efficacy of these NRF2 inducers has been verified on several FXN deficient models [81,85,135], where they significantly increased NRF2 mRNA levels [85], re-balanced the GSH/GSSG ratio [85,135], and up-regulated the FXN gene expression [85,135,136,137] (Figure 2).

Currently, clinical FA trials are focused on improving mitochondrial function and reducing oxidative stress [138]. Idebenone (Raxone^®^/Catena^®^), for instance, is proven to be effective on the mitochondrial function. But, despite an initial optimism on its cardiac impact, the neurological benefit in FA is still under evaluation [139,140,141]. RTA408 (Omaveloxolone), a specific NRF2 inducer [142], is currently being tested in a 12 months placebo-controlled trial (www.clinicaltrials.gov). EPI-743 (vatiquinone), another highly promising drug for FA [143], was approved in 2011 for children with genetically confirmed inherited respiratory chain diseases, but still not clinically tested in FA, although it has been proven to activate NRF2 and increase the expression of FXN in fibroblasts of FA patients [85].

### 4.2. X-Fragile (FXS)

FXS syndrome is caused by the absence or deficiency of FMRP, the gene product of *FMR1* [144]. In most cases, CGG trinucleotide expansions at the *FMR1* promoter determine its transcriptional silencing, giving rise to the disease [145]. More than 200 CGG repeat expansions determine the FXS phenotype, while premutation alleles, bearing 55–200 CGG tracts, are recognized to cause FXTAS. Mutations or deletions in the *FMR1* gene have been also reported in FXS patients [146,147], as well as mosaicisms of repeat length (i.e., some cells harboring the full mutation in one allele and others containing the premutation allele), or of methylation [148]. FXS represents the most common form of inherited intellectual disability [145], affecting 1:5000 males and 1:4000–8000 females [144], often associated with autism spectrum disorder comorbidities, estimated in about 50% patients [149]. Clinical FXS manifestations include language development delays, hyperactivity, anxiety, and physical dysmorphic features [150,151]. Females’ phenotype is usually less severe, due to the presence of the second X chromosome that can restore FMRP expression in approximately 50% of cells [152].

FMRP is a RNA-binding protein forming a ribonucleoproteic complex that associates to polyribosomes and regulates mRNA metabolism, acting as a translation suppressor [153]. Its expression is particularly high in neurons, where it shuttles between nucleus and axons or dendrites [154,155] and carries out its function, especially at postsynaptic sites, where it transports mRNA cargos [156,157] and where its activity is tightly regulated by synaptic receptors [158,159,160,161,162]. The absence of FMRP mostly determines an increase of translation rate of its targets [163,164,165,166] and impairs the synaptic development and plasticity in specific brain areas [158,167].

Oxidative stress in FXS has been suggested by a number of indirect evidences: (i) aging-dependent oxidative stress increase is responsible for impairments in long-term (LTP) potentiation in the hippocampus [168], a condition described in FXS [158,169]. (ii) Anxiety and autism, which are strongly linked to ROS impairments, are common conditions in the pathology [170,171,172]. (iii) As in Alzheimer’s disease, where oxidative stress is a recognized contributing factor in progression and pathogenesis of the disorder [48], in models of FXS amyloid beta (Aβ) expression is increased, probably as a consequence of FMRP-mediated dysregulation of amyloid precursor protein (APP) translation [173]. Despite these evidences, only a moderate increase of oxidative stress has been documented in FXS. Biochemical markers of oxidative stress have been detected in the brain of Fmrp1 KO mice, where increased ROS levels, high content of lipid peroxidation byproducts (i.e., thiobarbituric acid reactive substances, TBARS), enhanced carbonyl protein content, and imbalance of the GSH/GSSG redox ratio have been found in 2–4 months old mice, although these markers appeared to normalize over time [174]. Moreover, although FMRP favors the translation of SOD1 mRNA [175,176] and, consequently, Fmr1 null mice display a strongly reduced expression of SOD1 [176], to date, definitive proofs on oxidative stress depending on SOD1 decreased activity are still lacking in FXS. These apparent inconsistencies could be related to an indirect redox regulation operated by FMRP on NRF2. Indeed, the lack of FMRP increases the synthesis and the activity of the small Rho GTPase RAC1 [177], which exerts a role in ROS modulation and in the inflammatory response [178] by inducing NRF2 and up-regulating HO-1 expression [177]. RAC1 is known to participate in the activation of PI3K and MAPKs signaling pathways [179,180], thus suggesting that the modulation of NRF2 activity may depend on the RAC1 mediated AKT/PI3K-induced inhibitory phosphorylation of GSK3β [89,181], possibly rebalancing oxidative and inflammatory responses in FXS [177].

## 5. Oxidative Stress in CAG/polyQ Diseases

### 5.1. Spinobulbar Muscular Atrophy (SBMA)

SBMA, also known as Kennedy’s disease, is a neuromuscular X-linked disorder, which exclusively affects adult males [182]. Patients display progressive cramps, fasciculation, tremor, and weakness of skeletal muscles (especially bulbar, facial, and limb ones) [183], together with a mild androgen insensitivity syndrome (AIS) and sensory loss [184,185]. SBMA is due to spinal and bulbar motor neurons death, triggered by an expansion of more than 38 glutamine residues in the androgen receptor (AR) protein [186], a ligand activated transcriptional factor that mediates the cellular effects of the sex hormone testosterone and its metabolites [187]. The pathogenic mechanism in SBMA is unique respect to the other polyQ diseases. Nuclear and cytoplasmic inclusions of poliQ AR, UPS components, heat shock proteins (HSPs), and AR coactivators can be found in SBMA [188,189], however, other mechanisms participate to the pathogenicity of this disorder. PolyQ AR retains most of its functionality, but the region in which glutamine expansion occurs localizes in the N-terminal domain that is responsible for multiple protein–protein interactions [190]. This lead to partial loss of transcriptional activity [191], sequestration of transcription activators [192], and block of the AR-dependent non genomic signaling cascades [189]. Moreover, the pathogenic activity of polyG expanded AR seems to heavily depend on its ligands. Indeed, females homozygous for glutamine expanded AR display mild sings of SBMA [193] and studies in SBMA animal models demonstrated their androgen dependence [194,195]. Moreover, it has been proposed that WT AR signaling could play a role in the regulation of the expression of neurotrophins and growth factors known to support neuronal survival [182,196], a function that should be lost in the mutant receptor. Low expression of GDNF, for instance, was encountered in SBMA patients [197] and GDNF, IGF-1, and VEGF reduced expression was assessed in mouse models of the pathology [198,199].

Part of the SBMA pathogenic mechanism could be explained by increased ROS levels [200] and mitochondrial dysfunction [201]. Under physiological conditions, AR regulates the expression of several nuclear- and mitochondrial-DNA encoded proteins [202]. Thus, in cells expressing polyQ AR, numerous genes implicated in mitochondrial function are altered [200]. Moreover, mutant AR has been found to associate with the mitochondria in MN-1 cells, where it activates the intrinsic apoptotic pathway [200], and, in HeLa cells transfected with AR receptor bearing 48 glutamine expansions, mitochondria were sequestered in polyQ AR inclusions [203]. Although the toxicity of cytoplasmic AR aggregates has been questioned, [204,205] and mitochondrial sequestration was not found in polyQ AR expressing NSC34 motor neurons [206], mutant AR aggregates-mediated impairment of mitochondria transport along the neurites has been hypothesized in SBMA [206]. Another cause of mitochondrial impairment could be the androgen-dependent association of polyQ AR with the respiratory chain enzyme cytochrome c oxidase subunit Vb (COXVb), that could be trapped in mutant AR aggregates, interfering with the physiological function of oxidative phosphorylation [207]. It is important to underline that numerous cellular antioxidants are downregulated in SBMA, including proteins belonging to the pathway regulated by NRF2 (SODs, catalase [200], NQO1, and GPX). The expression of NRF2 itself was deeply reduced in motor neurons of mice carrying 100 glutamine expansions in AR with respect to the non-pathogenic 20 polyQ repeats-containing AR mice [208], and in MN-1 cells expressing the mutant AR [200]. Interestingly, some studies attempted to correct protein misfolding-induced aggregates by using curcumin, an antioxidant polyphenol whose activity was neuroprotective [209,210]. Curcumin treatment has been demonstrated to slow the protein aggregation [211] and to induce NRF2 activity [212,213]. A curcumin analog, ASC-J9, tested in cells and in a mouse model of SBMA, demonstrated beneficial effects on mutated AR aggregates, motor impairments, muscular atrophy, and VEGF expression [214]. Another curcumin analog, ASC-JM17, has been tested on cultured SBMA cells and has been found to activate NRF2 and its signaling pathway, determining the transcription of its target genes, including NQO1, HO-1, catalase, and GCL [215]. These evidences support a promising role for this class of molecules to reverse multiple SBMA pathogenic defects.

### 5.2. Huntington’s Disease (HD)

Huntington’s disease (HD) is a progressive, autosomal dominant neurodegenerative disease with defects in the striatum, cerebral cortex, and thalamus [216,217]. The HD disorder is caused by the abnormal expansion of the nucleotide triplet CAG in the gene coding for the protein huntingtin [218]. In the huntingtin gene (HTT) of healthy subjects, the number of trinucleotides CAG repeats varies from 1 to 34, while in HD patients, the CAG triplet expansion ranges between 35–140 repetitions [219]. Clinical features of HD include progressive motor dysfunction, psychiatric disturbance, cognitive decline, dystonia, bradykinesia, and dementia, ultimately leading to death within approximately 15–20 years from the age of onset [220]. The genetic abnormality in the HD gene leads to the formation of a mutant huntingtin protein (mHtt), which is normally involved in the vesicle transport and represents a scaffold for the autophagic machinery [221,222]. The mutant protein exhibits toxic properties, leading to protein aggregation, transcriptional dysregulation, defective energy metabolism, chronic inflammation, and oxidative stress [223,224,225,226]. Inflammation, mitochondrial dysfunction, and oxidative stress are some of the key pathways persistently abnormal in mouse models of HD and in autoptic tissues of patients.

Several pharmacological HD mice models have been developed, resembling defective neuro-motor functions described in human HD patients [227,228,229,230] and supporting oxidative damage as a pathogenic mechanism underlying neurodegeneration in this disease [231,232]. Increased markers of oxidative stress, mitochondrial failure, and chronic inflammation have been found in brain tissue of HD patients. High levels of malondialdehyde, 8-hydroxy-deoxyguanosina, and carbonyls, and lower levels of GSH, SOD1, and GPX, have been detected in plasma and red blood cells of patients [233,234]. Additionally, mitochondrial DNA damage, low levels of oxidative phosphorylation enzymes, and iron-mediated mitochondrial impairment have been shown in autoptic brain tissues of patients [231,235]. In addition, increased amounts of circulating pro-inflammatory cytokines have been reported in patients, whose levels correlated to the severity of the disease [234]. Numerous studies have been focused to reduce oxidative damage in HD by using antioxidants (alpha-tocopherol, CoQ10, vitamin E, vitamin C, N-acetylcysteine (NAC), lipoic acid [236,237,238,239,240,241,242]). Nevertheless, these compounds have shown a moderate effectiveness in counteracting oxidative stress in mouse models, thus leading to hypothesize that a pharmacological upstream activation of NRF2 should be required. Recently, the potent NRF2 inducer SFN has been tested, showing increased mHtt degradation and a significant reduction of cytotoxicity by the NRF2-mediated activation of the ubiquitin–proteasome system [243]. The SFN pre-treatement ameliorated behavioral impairments and reduced pro-inflammatory cytokines in the striatum of a 3-nitropropionic acid (3-NP) mouse model by attenuating neuroinflammation and oxidative stress [243]. High susceptibility to oxidative stress has been also found in human HD neural stem cells, where the genetic correction of the disease-causing mutation restored the redox balance [244]. The protective effect of NRF2 activation in HD patients has been further confirmed in primary monocytes, where the NRF2 induction inhibited the release of pro-inflammatory cytokines (IL-6, IL-1, IL-8, and TNFα) [244]. As in other neurodegenerative diseases, also in HD has been hypothesized a role for ferroptosis in the pathogenic mechanism, mainly as a consequence of increased iron levels that were detected in brain regions of patients [235,245]. Therefore, even in HD, NRF2 can represent a strategic therapeutic target, for its ability in preventing iron overload and regulating ferroptosis-related genes expression [246]. DMF, for instance, exerted beneficial effects on survival and motor functions in R6/2 and YAC128 models of HD, preserving the neuronal integrity in striatum and motor cortex, and slowing degeneration [247]. Additionally, gintonin (GT), a ginseng-derived lysophosphatidic acid receptor ligand, was effective on the NRF2 pathway in the striatum of 3-NPA mice, by protecting the mitochondrial function and reducing the expression of inflammatory mediators (cytokines, COX-2, and iNOS) [248].

### 5.3. Spinocerebellar Ataxias (SCAs)

SCAs comprise more than 40 disorders, all characterized by progressive degeneration of the cerebellum, which determines abnormal coordination and gait impairments [249]. Clinical features have been used to initially subdivide these disorders. Indeed, almost all forms of SCAs are characterized by cerebellar degeneration, with involvement of Purkinje and granule cell layers and neurons of deep cerebellar nuclei [250,251]. However, other regions of the brain can be affected. In all polyQ SCAs, for example, with the exception of SCA6 where Purkinje neurons are exclusively implicated, brainstem is involved [251,252]. Moreover, many forms of SCAs share clinical signs on basal ganglia, spinal cord, cerebral cortex, and peripheral nerves, while epilepsy is restricted to SCA10, and pigmentary retinal degeneration only occurs in SCA7 [251,252,253]. A genetic classification has been developed for these disorders based on heritance (i.e., autosomal dominant, autosomal recessive, X-linked, and mitochondrial) [254] and type of mutation (microsatellite repeat expansion or point mutation [255]. Despite this, the gene or mutation responsible for many SCAs are currently unknown [253]. The pathogenic mechanism appears variegated even among the autosomal dominant forms of SCA. Indeed, while polyQ tracts encoded by CAG repeats are present in at least 7 disorders (SCA1, 2, 3, 6, 7, 17, and DRPLA), tri- penta- or hexanucleotide expansions, located in introns or in the 3′UTR, occur in SCA8, 10, 12, 31, 36, and 37, determining an unclear pathogenic mechanism compatible with the generation of toxic mRNA or transcriptional silencing [18,255]. Only the polyQ SCAs were considered in this review, being the best characterized for their oxidative stress defects. PolyQ SCAs display a wide range of phenotypes due to the great instability of repeat expansion length, also responsible for the clinical anticipation reported for these disorders. This is most evident in the Machado–Joseph disease (SCA3), where the pathologic onset can occur in childhood, in middle-age, or have a late onset, depending on the expansion length. In people with smaller repeats, the disease can present as restless leg syndrome [251,256]. The physiological functions of the proteins responsible for SCAs are highly heterogeneous: ataxin 1, the protein mutated in SCA1, interacts in the nucleus with transcriptional regulators and with the splicing complex [257]; similarly, ataxin 7, TATA-binding protein (TBP) and atrophin 1, whose mutations are the underlying cause of SCA7, SCA17, and DRPLA, are involved in transcriptional regulation processes, but with different roles (e.g., activating or repressing function) and with different interactors [258,259,260]; ataxin 2 (SCA2) regulates translation, by the interaction with poly(a)-binding protein [261]; ataxin 3 (SCA3) is a de-ubiquitination enzyme [262]; Cav2.1α1A (SCA6) is a subunit of the voltage dependent calcium channel [263].

Despite the functional heterogeneity and beside the partial loss of ataxin 1 function observed in SCA1 [264], in all these disorders, the proposed pathogenic mechanism is the same: polyQ expansions promote protein misfolding that results in aggregation [253]. This leads to defective interactions with common partners and determines impairment in shared cellular pathways leading to cerebellar neurons degeneration [265]. The aggregates grow by time, forming big inclusions that may represent a valid biomarker for the disease. However, a discrepancy has been evidenced, as the presence of protein aggregates has been often reported to correlate with neuronal survival rather than to cell death [251]. One hypothesis is that small CAG-expanded protein oligomers, produced at earlier steps of aggregation, are more toxic for the cell than larger ones [251]. As reported for other polyQ diseases, these inclusions are positive for the presence of transcription factors [192], as well as for proteins belonging to the quality control assessment (i.e., chaperons) and to the UPS system [266,267], suggesting that a major pathogenic route in these disorders consists in the derangement of the two cellular systems functioning in the clearance of damaged proteins [268]. In line with this, some studies reported that both polyQ Ataxin 1 and 7 fail to be degraded by the proteasome, as it occurs in normal conditions [269,270]. Moreover, due to its localization near the polyQ expanded tract, the function of the Jospephin domain, which mediates the de-ubiquitinating activity of ataxin 3, could be altered, impairing ataxin 3 association with the proteasome [271] and further exacerbating the activity of the UPS system [253].

Oxidative stress, mitochondrial impairments, and NRF2 involvement are widely reported in SCAs and, due to the fact that Purkinje neurons are among the most energy-demanding cellular types [272], it is not surprising that mitochondrial impairment in these disorders have been proposed to strongly contribute to the disease progression [273]. In the SCA1 mouse model, the mutant ataxin 1 has been found to sequester the high mobility group box1 complex (HMGB1), inhibiting its function [274] and determining the increase of mitochondrial DNA damage [275]. Successive studies performed in Sca1^154Q/2Q^ mice evidenced morphological mitochondria alterations, dysfunctional electron transport chain (ETC) enzyme activities, and increased oxidative damage [273]. Importantly, in young Sca1^154Q/2Q^ mice, that have not yet exhibited mitochondrial defects, the treatment with a quinone, which is able to modulate NRF2 nuclear translocation and activity (MitoQ) [276], is capable of delaying the onset of motor coordination defects [273]. Likewise, MitoQ administration to mice already displaying the pathologic deficits improves mouse phenotype [273]. In cultured SCA2 patient’s fibroblasts, dysfunctions of mitochondrial network structure, alterations of antioxidant genes transcription and expression, increases of O^−^ and H_2_O_2_ production, and impaired activities of ETC complex I, II, and III have been further evidenced [277], strongly connecting mitochondrial defects to ROS overload and oxidative stress. Despite many of these defects are rescued by treating SCA2 fibroblasts with CoQ_10_ [277], its clinical effect on a cohort of SCA1, 2, 3, and 6 patients is unclear. The drug administration was effective on clinical baseline outcome only in SCA1 and 3 patients, but it did not modify the progression of the disease in the 2 years follow-up trial [278]. Among SCAs, SCA3 is the most common [279] and the best characterized regarding the relationship among ROS, NRF2 imbalance, and neurodegeneration. In cells stably transfected with CAG expanded ataxin 3, a strong reduction of the GSH and GSH/GSSG ratio was observed. Paired to this, reduction of glutathione reductase (GSR), SOD, and catalase activities was reported, determining an increase of mitochondrial DNA damage, also assessed in SCA3 patient’s blood [280]. Reduction of ETC complex II activity is reported in different SCA3 cellular and mouse models and in SCA3 human lymphoblastic cell lines, supporting an increase of ROS generation [281]. The redox imbalance has been demonstrated also in patients. A significant reduction of thiols levels, which include GSH and thioredoxins, has been described in a case-control study enrolling 7 patients with SCA3, although total polyphenols, lipid peroxides, and ROS levels appeared unchanged [282]. Nevertheless, a recent study performed on a larger number of SCA3 patients attested to an increase of ROS levels and the inhibition of SOD, with a parallel reduction of GPX activity that correlated with disease severity [283]. Notably, all these parameters were close to physiological values in the pre-symptomatic group of SCA3 patients [283].

**Figure 3:**
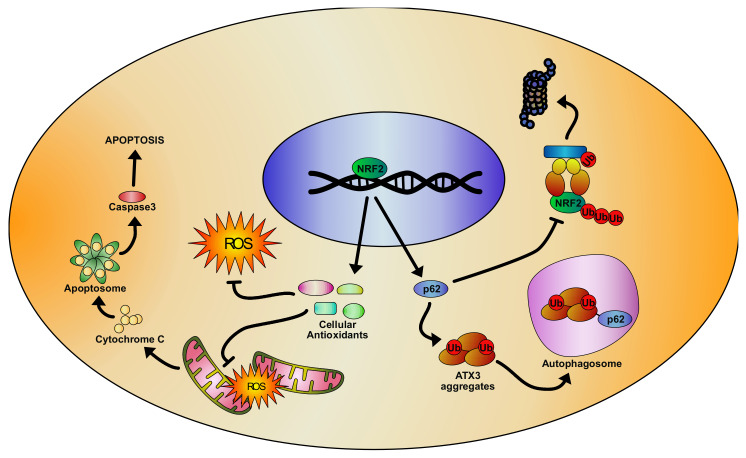
Representative model of the NRF2 signaling pathway activation in spinocerebellar ataxia 3 (SCA3), based on literature evidences. As a consequence of NRF2-mediated activation of the antioxidant response, reduction of cellular and mitochondrial ROS production is observed, thus inactivating the apoptotic pathway. In addition, NRF2 increases cellular levels of p62, which shuttles the mutant ataxin 3 aggregates to the autophagosomes, reducing their cellular concentration. At the same time, p62 interferes with the KEAP-1/NRF2 complexes, thus blocking the KEAP-1 mediated NRF2 degradation and sustaining its activity.

Mutated ataxin 3 activity is connected with oxidative stress impairments in SCA3. Oxidative stress induces the nuclear translocation of ataxin 3 and this occurs both in normal and pathological conditions [284]. However, this acquires a particular importance in the pathologic context, as CAG-expanded repeats-containing proteins exert their toxic effects predominantly at nuclear level [285,286,287,288,289]. Because ROS production increases with age [290], the toxic effect could progressively worsen by time and increase neuronal death. Moreover, ataxin 3 has been found to interact with forkhead box class O 4 (FOXO4), a transcription factor implicated in the regulation of cell response to stress stimuli, including oxidative stress [291]. Under physiological conditions, ataxin 3 binding to FOXO4 is a necessary pre-requisite for SOD2 transcription. Although both normal and mutated ataxin 3 are able to interact with FOXO4, only the wild type protein can activate the FOXO4-dependent binding at SOD2 promoter. As a consequence, SOD2 expression is reduced in SCA3 cells, thus contributing to increase oxidative stress and cytotoxicity [292]. Several studies demonstrated reduced NRF2 levels in HEK293 and SH-SY5Y, after transfection with ataxin 3 mutant cDNA. The expression of NRF2 downstream targets were also reduced, thus determining a further increase of ROS [293,294]. Cell treatment with plant-derived antioxidant compounds were able to rescue these biochemical defects by triggering NRF2 activation and enhancing the expression of NQO1, GCL, GST, and SOD2 [293,294]. Importantly, the NRF2 activation, or its overexpression, reduced the aggregation of mutant ataxin 3 and the activation of the caspase3-dependent apoptotic pathway, while silencing the NRF2 expression led to increased ataxin 3 aggregates formation [294]. The administration of resveratrol and caffeic acid to mutant ataxin 3-expressing human SK-N-SH neuroblastoma cells and to a drosophila model of SCA3 enhanced the NRF2 activity, up-regulating the expression of NQO1, catalase, HO-1, GPX, SOD, and GR and causing a consistent reduction of total and mitochondrial ROS [295]. In parallel, the treatment was also able to induce the NRF2-mediated expression of autophagy-related proteins (i.e., p62), leading to decreased expression of mutant ataxin 3 and its aggregates (Figure 3), ultimately determining an extension of mutant flies life span [295].

The link between oxidative stress-induced damage and polyQ mutant protein aggregation was investigated also in SCA7, with similar results obtained in the studies described above. In PC12 cells, stably expressing a mutant ataxin 7 bearing 65 Q residues, oxidative stress is increased, with a parallel reduction of GSH content and dysregulation of catalase, SOD1, and GST protein levels, due to the aberrant activation of the NADPH oxidase (NOX) complex [296]. These defects were ameliorated by the use of two antioxidants, N-acetyl cysteine (NAC) and vitamin E, both known to induce NRF2-dependent transcription [72,85]. Both compounds were able to reduce the level of mutant ataxin 7 aggregation [296]. Notably, by overexpressing SOD1 or RORα, a transcriptional factor that activates the anti-oxidant gene transcription [297], a comparable reduction of mutant ataxin 7 aggregation was observed, further confirming the link between oxidative stress and pathologic polyQ protein [296]. In a mouse model of SCA17, the transcriptional dysregulation induced by CAG expanded TBP determines the downregulation of the heat shock protein beta1 (HSBP1) [298], a protein known to protect cells from oxidative stress in HD [299]. In addition, lymphoblastoid cells obtained from patients with SCA17 resulted highly susceptible to oxidative stress and to the ROS-induced cell death [300]. Proteomic analysis performed on SCA17 lymphoblastoid lines confirmed the involvement of the NRF2 signaling pathway in the disease, as evidenced by NQO1 and HO-1 different expression patterns in cells carrying mutation [301]. The treatment with two NRF2 inducers (resveratrol or genipin) [72,302] was able to rescue the antioxidant genes transcription defects and to low ROS generation [301]. Lastly, the implication of oxidative stress in the DRPLA pathogenic mechanism is poorly investigated to date, however, DNA and RNA oxidative by-products have been found in patients, particularly in a subset of cases showing progressive myoclonus epilepsy (PME) [303]. The SOD2 expression was reduced in 70% of examined patients and this correlated with clinical symptoms of epilepsy [303].

## 6. Oxidative Stress in RNA Gain of Function Expansion Disease

### 6.1. Fragile X–Associated Tremor Ataxia Syndrome (FXTAS)

While a number of CGG repetitions greater than 200 in the FMR1 gene determines FXS, CGG triplets ranging between 55–200 in the fragile X locus, a condition known as pre-mutation state, gives rise to FXTAS [304,305]. FXTAS is a neurodegenerative disorder principally occurring in adult male carriers (50 years or more) and whose penetrance increases with age [305,306]. FXTAS clinical features include late onset and progressive cerebellar gait ataxia and intention tremor, with associated parkinsonism, cognitive deficits, and peripheral neuropathy [43,305,306,307]. Individuals developing FXTAS have normal or relatively low FMR1 protein levels, but increased FMR1 mRNA transcript [308], which accumulates in the nucleus of neurons and astrocytes in ubiquitin-positive inclusions [309,310]. Long CGG expansions in FMR1 mRNA are able to sequestrate numerous proteins that co-localize with the pathologic intranuclear inclusions in FXTAS animal models and patients [311,312,313,314]. FXTAS CGG expanded intranuclear inclusions are dynamic and form structures capable to trap a variety of proteins over time [47]. One of the first proteins to be sequestrated is the splicing factor SAM68 [47], a critical regulator of alternative splicing and polyadenylation in the nervous system [315,316,317]. Nevertheless, a recent work demonstrated that nearly 200 proteins can be found in FXTAS intranuclear inclusions, 36% of them being RNA binding factors, with a strong enrichment of SUMO2, ubiquitin, and p62 proteins, indicating that aggregates are mostly composed of proteins tagged for degradation [314].

Oxidative stress is well known in FXTAS [318,319,320,321] and proteins belonging to redox response have been identified as SUMO2/3 substrates in patients, thus suggesting to be sequestrated in aggregates [314]. A number of mitochondrial dysfunctions has also been reported. A significant decline of oxidative phosphorylation (OXPHOS), increase of lipid peroxidation [318], oxidative biomarkers, and ROS [321] have been evidenced in fibroblasts and blood of FXTAS patients. Abnormal expression and function of mitochondrial proteins were further reported in patient’s brain samples [320,322]. Because of this, oxidative stress has been proposed to participate to the FXTAS pathogenic formation of nuclear aggregates. By this model, a ROS-induced increase of oxidized proteins could exceed the UPS degradative capacity and lead to the accumulation of ubiquitin- and SUMO2/3-tagged proteins with mRNA molecules of FMRP1. In late stages, also the p62-mediated shuttling of aggregates towards autophagosomes would be abrogated, for the excessive enlargement of the inclusions, thus ultimately leading to the nuclear p62 trapping [314].

Some clues suggest the involvement of the NRF2 signalling pathway in this pathology. (i) Reduced expression of mnSOD, which is a NRF2 target gene, was attested in FXTAS [320]. (ii) Alterations of the mitochondrial network in patients [318] and impairments in their density and transport dynamics in mice carrying CGG premutation have been reported [323], both suggesting impairments in cytoskeletal proteins responsible for mitochondrial distribution and cell morphology [324]. These defects resemble the altered NRF2 trafficking observed in FA, where the increase of oxidative stress impairs cytoskeletal organization [325] by causing the mislocalizing of KEAP1-NRF2 complexes, normally bound to actin filaments [326], and leading to the failure of NRF2-mediated transcriptional activity [117]. (iii) As in FA, also in premutation carriers, FXN expression is low [322]. The reduced expression of the zinc transporter ZnT6 alters the zinc availability in FXTAS, impairing its incorporation, the mitochondrial processing peptidase (MPP), and the mitochondrial intermediate peptidase (MIP), two Zn-dependent proteases responsible for the maturation of mitochondrial proteins [327], including FXN [328,329]. The consequent increase of the premature form of FXN, in respect to the mature one, leads to defective iron metabolism and oxidative stress [322], and suggest that the similarities between FA and FXTAS could also be extended to NRF2 signalling pathway.

### 6.2. Myotonic Dystrophy (DM)

DM is an autosomal dominant disorder, which arises from 2 different mutations: DM1, determined by 50-1000 CUG triplets in the 3′UTR of *DMPK* gene [18,41] and DM2, caused by 75–11000 expansions of the tetranucleotide CCTG in the first intron of *ZNF9*. [18,42]. DM1 and DM2 are multisystemic diseases sharing a common symptomatology characterized by myotonia, muscular dystrophy, cardiac defects, cataracts [330], and neurological manifestations [331,332]. Unlike DM1, DM2 does not show congenital forms [333]. DM shows a marked somatic instability of repeat expansions that, in DM1, are reported to increase of about 50–80 repeats per year and, in DM2, appear to be even more pronounced [330,334,335]. Depending on the repeat length, the severity of DM1 and the onset of the pathology range from “mild” manifestation (baldness and cataracts) to a “classic” or “juvenile” form, with worse symptoms [19]. On the contrary, although the same clinical heterogeneity is observed in DM2, the pathologic onset and disease severity do not seem to depend on the size of expansions in this disorder [336]. Clinical anticipation, prominent in DM1 [337], appears mildly in DM2 [338].

Different hypotheses have been proposed to explain the pathogenic mechanism in DM. Early studies suggested that the pathological defects observed in DM1 could be determined by the decrease of DMPK expression, mediated by CUG expansions [339] and/or by the trans-acting effect of the expanded mRNA, able to reduce the processing of WT DMPK mRNA [340]. Clinical similarities led to support a common pathogenic mechanism for DM1 and DM2 and, to date, an RNA toxic gain of function is the most credited. In particular, both CUG triplet containing DMPK mRNA and spliced ZNF9 intron1 containing long CCTG sequences are able to sequestrate in the nucleus the splicing factors MBNL1 and 2 [341,342] and, at the same time, to raise the RNA binding activity of CUG-binding protein 1 (CUG-BP1 or CELF1) [330,343]. This changes the cellular alternative splicing output, determining the defects observed in the disease [46,344].

**Figure 4:**
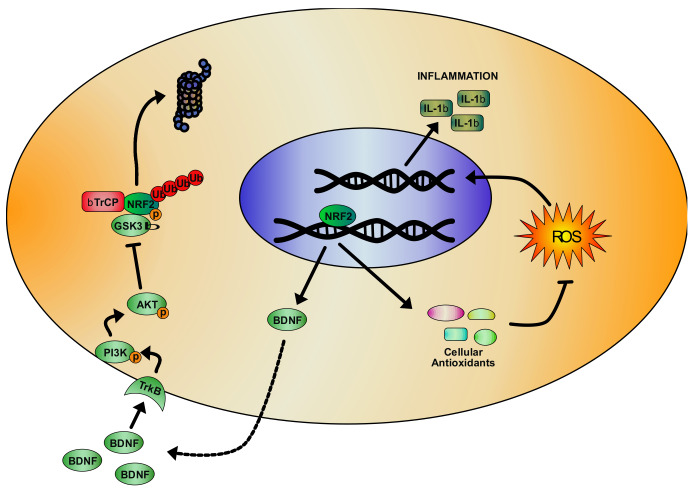
Representative model of the NRF2 signaling pathway activation in myotonic dystrophy 1 (DM1), based on literature evidences. Brain-derived neurotrophic factor (BDNF) activation of PI3K/AKT pathway determines the inhibitory phosphorylation of GSK3β by blocking NRF2/KEAP-1-indipendent degradation. As BDNF is a NRF2 target, this can start a positive feedback contributing to NRF2 activation. At the same time, the transcription of NRF2 antioxidant target genes reduces oxidative stress in DM1 cells and the pro-inflammatory cytokine IL-1β levels.

Many of the clinical features showed in DM, including myotonia, progressive muscle weakness, cataracts, frontal alopecia, and cognitive decline, suggest an increased susceptibility to oxidative stress in this pathology, as observed in premature and accelerated aging [345]. While in DM2, oxidative stress is still poor investigated, a pathogenic involvement of ROS has been evidenced in DM1. Increased sensitivity to oxidative stress and strong activation of the pro-apoptotic p38 and JNKs pathways have been reported in the C2C12 cell line transfected with human mutant MDPK containing a variable number of CTG repeats [346]. On the contrary, in cells having only 5 CTG repeats, ERKs were preferentially activated [347]. Moreover, studies performed in DM patients have demonstrated an increase of lipid peroxidation and ROS levels, with a parallel decrease of the antioxidant CoQ_10_ content [348]. Increased oxidative stress and ROS-induced inflammation are known to produce cognitive dysfunctions [349,350] and depressive behaviours [351,352], conditions observed in the MBNL2 KO mouse model of DM1 [353]. In these mice, the chronic administration of methylphenidate (MPH) was able to partially rescue the cognitive defects and depressive-like behaviours, and to reduce the reactive microglia and pro-inflammatory cytokine IL-1β levels [353]. The treatment with MPH was shown to increase NRF2 gene expression in the hippocampus of MBNL2 KO mice and the brain-derived neurotrophic factor (BDNF) levels, which regulates NRF2 nuclear translocation by means of an ERK/PI3K-dependent activation [354]. These findings suggest that the rescue of behavioural defects in MBNL2 KO mice may depend on NRF2-mediated reduction of oxidative stress and inflammation. In line with this, it is important to note that in NRF2 KO mice, an increase of the serum level of pro-inflammatory cytokines and a decrease of the BDNF expression have been reported in association to a depressive-like phenotype [355]. In the same way, NRF2 activation is able to reduce depression and serum content of pro-inflammatory markers induced by lipopolysaccharide (LPS) injections in mice [356]. Cognitive defects [331,332] and depression [357], together with serum increased concentration of the pro-inflammatory IL-6 [358] have been found in DM1 patients, thus the pharmacological NRF2 induction could be very promising in this disease (Figure 4).

## 7. Conclusions

Around the first years of the 1990s, the discovery of a new type of unstable mutation, the expansion of DNA microsatellite repeats, was found as the underlying cause of FXS, DM, and SBMA [359], helping to understand the molecular basis of the clinical anticipation concept in heritable diseases and allowing the definition of a new class of disorders, characterized by the earlier onset and severity in succeeding generations. Since then, this class has expanded, making the current subdivision, which accounts for the general pathogenic mechanism (i.e., loss/gain of function of protein or mRNA), necessary. As, to date, DNA expansions are known to cause more than 30 disorders (16), it is somehow surprising that oxidative stress is involved in a major part of such a vast group of pathologies. At the same time, mounting evidence points at ROS imbalance as a common thread in neurodegeneration and a plethora of neurodegenerative conditions share oxidative-related defects [48,49]. Cellular pathways in charge of regulating the oxidative stress defenses are often deregulated in DNA expansion disorders, providing an inadequate response to ROS overload. In line with this, the NRF2-ARE axis has been found to be defective in most of the pathologies we reviewed here and, in some of these, beneficial effects have been observed by modulating NRF2 signaling (Table 1). Therapeutic interventions aimed at re-establishing the NRF2 pathway can be very promising to ameliorate the patient’s condition by rescuing oxidative stress-induced defects. Notably, NRF2 induction has been demonstrated to partially recover the primary defects in FA [85,135,136,137] and in SCA3, where it mediates the reduction of ataxin 3 aggregates [294,295], thus paving the way for NRF2-targeted therapies.

## Author Contributions

Conceptualization: P.L.R, F.P.; writing of the Huntington’s disease and Friedreich’s ataxia sections: S.P.; writing & editing: P.L.R., E.S.B., F.P.; figures: P.L.R. All authors have read and agreed to the published version of the manuscript.

## Figures and Tables

**Table 1:** Table summarizing findings on antioxidant drugs/NRF2-activating compounds and their main effects in DNA repeat expansion disorders.

Disease	Compound	Model	Effect of Treatment	Ref.
FA	SFN, DMF, NAC, EPI-743, RTA408, Idebenone	FA patients’ fibroblasts	Increase of GSH content; enhancement of *FXN*, NRF2 and down-stream genes mRNA.	[111]
	SFN, DMF	shFXN NSC34 motor neurons	Rebalance of GSH/GSSG ratio; increase of FXN, NRF2 and down-stream genes expression.	[124]
	Idebenone	Patients	Reduction of cardiac hypertrophy.	[128]
	EPI-743	Patients	Improvement of neurological functions.	[132]
SBMA	ASC-J9	AR-112Q PC12 cells; AR-97Q mice	Reduction of AR aggregates; rescue of motor defects and muscular atrophy; increase of VEGF expression.	[203]
	ASC-J17	SBMA patients’ fibroblasts; AR97Q mouse; AR52Q drosophila	Increase of NRF2 down-stream genes; suppression of polyQ toxicity in mutant flies; amelioration of mutant mice phenotype and decrease of mutant AR accumulation.	[204]
HD	SFN	mHtt-94Q Hek293	Increase of mHtt degradation and reduction of mHtt-induced toxicity.	[232]
	MIND4-17	HD patients’ primary monocytes	Reduction of inflammatory cytokines expression.	[233]
	DMF	R6/2 and YAC128 mice	Increased survival and motor functions; preservation of striatal neurons morphology; increase of NRF2 expression.	[236]
SCA1	MitoQ	Sca1 154Q/2Q mice	Improvement of motor coordination defects; reduction of mitochondrial morphological abnormalities and ETC activity defects.	[262]
SCA3	*Glycyrrhiza inflata* extract, AMGZ, Licochalcone A	ATXN3/Q75-GFP Hek293 and SH-SY5Y cells	Decrease of Ataxin3 aggregates; up-regulation of NRF2 and down-stream genes; reduction of GSSG and ROS levels.	[282]
	*Gardenia jasminoides* extract, genipin, geniposide, crocin	ATXN3/Q75-GFP Hek293 and SH-SY5Y cells	Reduction of Ataxin3 aggregates and Caspase3 activity; increase of NRF2 and its target genes; decrease of ROS concentration.	[283]
SCA17	Resveratrol, genipin	SCA17 lynfoblastoid cells	Increase of NRF2 antioxidant target genes and cell viability; decrease of ROS.	[290]
DM1	MPH	Mbnl2 KO mice.	Increase of *NRF2* and *BDNF* expression; rescue of behavioral deficits; decrease of inflammation.	[344]
